# Epidemiology and costs of dengue in Thailand: A systematic literature review

**DOI:** 10.1371/journal.pntd.0010966

**Published:** 2022-12-19

**Authors:** Usa Thisyakorn, Surasak Saokaew, Elaine Gallagher, Randee Kastner, Rosarin Sruamsiri, Louisa Oliver, Riona Hanley

**Affiliations:** 1 Tropical Medicine Cluster, Chulalongkorn University, Bangkok, Thailand; 2 Center of Health Outcomes Research and Therapeutic Safety (Cohorts), School of Pharmaceutical Sciences, University of Phayao, Phayao, Thailand; 3 Unit of Excellence on Clinical Outcomes Research and IntegratioN (UNICORN), School of Pharmaceutical Sciences, University of Phayao, Phayao, Thailand; 4 Division of Social and Administrative Pharmacy, Department of Pharmaceutical Care, School of Pharmaceutical Sciences, University of Phayao, Phayao, Thailand; 5 Takeda Pharmaceuticals International AG, Zurich, Switzerland; 6 Takeda Vaccines Inc., Cambridge, Massachusetts, United States of America; 7 Takeda (Thailand) Ltd., Bangkok, Thailand; 8 Adelphi Values, Bollington, Macclesfield, United Kingdom; National University Singapore Saw Swee Hock School of Public Health, SINGAPORE

## Abstract

**Background:**

Dengue is the fastest-spreading vector-borne viral disease worldwide. In Thailand, dengue is endemic and is associated with a high socioeconomic burden. A systematic literature review was conducted to assess and describe the epidemiological and economic burden of dengue in Thailand.

**Methods:**

Epidemiological and economic studies published in English and Thai between 2011–2019 and 2009–2019, respectively, were searched in MEDLINE, Embase, and Evidence-Based Medicines reviews databases. Reports published by the National Ministry of Public Health (MoPH) and other grey literature sources were also reviewed. Identified studies were screened according to predefined inclusion and exclusion criteria. Extracted data were descriptively summarised and reported following the Preferred Reporting Items for Systematic Reviews and Meta-Analyses (PRISMA) guidelines.

**Results:**

A total of 155 publications were included in the review (39 journal articles and 116 grey literature). Overall, dengue incidence varied yearly, with the highest rates per 100,000 population in 2013 (dengue fever (DF) 136.6, dengue haemorrhagic fever (DHF) 100.9, dengue shock syndrome (DSS) 3.58) and 2015 (DF 133.1, DHF 87.4, DSS 2.14). Peak incidence coincided with the monsoon season, and annual mortality was highest for DSS, particularly in the age group 15–24-year-olds. The highest dengue incidence rates were reported in children (10–14-year-olds) and young adults (15-24-year-olds), irrespective of dengue case definition. Economic and societal burdens are extensive, with the average cost per case ranging from USD 41 to USD 261, total cost per year estimated at USD 440.3 million, and an average of 7.6 workdays lost for DHF and 6.6 days for DF.

**Conclusions:**

The epidemiological, economic, and societal burden of dengue in Thailand is high and underreported due to gaps in national surveillance data. The use of expansion factors (EFs) is recommended to understand the true incidence of dengue and cost-benefit of control measures. Furthermore, as dengue is often self-managed and underreported, lost school and workdays result in substantial underestimation of the true economic and societal burden of dengue. The implementation of integrated strategies, including vaccination, is critical to reduce the disease burden and may help alleviate health disparities and equity challenges posed by dengue.

## Author summary

Dengue is a vector-borne viral disease affecting over 100 million people globally. In Thailand, dengue is endemic, but the true estimation of the disease burden is underreported. A systematic literature search for epidemiological (2011–2019) and economic (2009–2019) studies was conducted to analyse the disease incidence, seroprevalence, serotype distribution, mortality, expansion factor, and direct and indirect costs at a country and regional level. Peak dengue incidence was highest in 2013 and 2015 and overlapped with the annual monsoon season. Children and young adults were the most affected population, with the highest case fatality rates found in young adults aged 15–24 years with dengue shock syndrome (DSS). Limited evidence indicates a high economic and societal impact of dengue. These estimates place dengue as a significant public health concern in Thailand and emphasise the need for introducing an integrated disease management programme including vaccination strategies.

## Introduction

Dengue is the fastest-spreading vector-borne viral disease affecting over 100 million individuals per year globally. Over 70% of the population at risk of the disease are in the Southeast Asia and Western Pacific Regions [1]. Dengue is recognised as a major public health problem in Thailand affecting people of all ages, with cases reported from all four regions: North, Central, Northeast, and South [1,2]. The epidemics in the country follow a cyclic pattern, with the number of reported cases ranging from 20,000 to 140,000 per year for the period 2000 to 2011 [3]. Temperature, rainfall, and humidity contribute to the transmission cycle of dengue resulting in seasonal peaks with high incidence during the rainy season [4,5].

Infection with a dengue virus (DENV) typically causes asymptomatic infection or mild to moderate febrile illness. Globally, a small proportion of patients (about 5%) develop severe dengue, which can be life-threatening [6]. In Thailand, an average of 0.016 deaths per 100,000 population was reported for the period between 2000 and 2011, with the highest case fatality rates among young children [3]. At the febrile stage, dengue can be misdiagnosed as other diseases, including Zika, chikungunya, and COVID-19, due to the overlapping symptoms encompassing fever; rash; eye, muscle, and joint pains; vomiting; and headache [7–10].

Dengue is a notifiable disease in Thailand [3]. A nationwide passive surveillance system subject to inherent limitations is used to record dengue cases, severity, and demographics of detected cases. Both active and sentinel surveillances are rarely performed [3,11]. The 1997 World Health Organization (WHO) dengue case definition, which classifies dengue into three categories: DF, DHF, and DSS is extensively used in Thailand [3,12]. DF, a mild form of dengue often presents undifferentiated febrile disease that can lead to fatal DHF and DSS if not appropriately treated [13,14]. All cases are reported within 24 hours at the district level and are further aggregated at the provincial level and by the Bureau of Epidemiology [3].

In 2019, WHO designated dengue as one of the top ten threats to public health and the disease is associated with significant societal and economic burdens [15]. The true cost of the disease is likely underestimated due to underreporting and misdiagnosis of cases and dengue-related deaths owing to the wide spectrum of clinical presentations, and reliance on passive surveillance systems [16,17]. In 2013, the economic burden of dengue in Thailand was estimated at USD 424 million annually, and the total unit cost per case in 2010 was estimated at USD 793.6 [17,18]. Limited access to healthcare facilities across different populations and socioeconomic status further contributes to the underestimation of the disease burden.

Given the high burden of dengue, understanding the epidemiological and cost trends in Thailand is essential for the allocation of health resources and the implementation of effective control and preventive strategies. A systematic literature review was conducted to assess and describe published data on the epidemiological and economic burden of dengue in Thailand and to update a previous literature review [3].

## Methods

The review was performed according to the Cochrane Handbook for Systematic Reviews guidelines [19] and the results are reported according to the PRISMA guidelines [20,21].

### Data sources and search strategy

To identify articles on the epidemiology and costs of dengue in Thailand, MEDLINE, Embase, and Evidence-Based Medicine reviews databases (Cochrane Database of Systematic Reviews, Cochrane Clinical Answers, Database of Abstracts of Reviews of Effects, and Health Technology Assessment) were searched via the OVID platform on 10 September 2019. Separate searches were conducted for epidemiological and cost burdens and restricted to articles published in English and Thai languages. The publication year was limited to 2011–2019 for the epidemiology burden, as a systematic literature review had already been conducted from 2000–2011 [3]. However, studies reporting data prior to 2011 and not captured in the previous systematic literature review were included in this review. For cost studies, the publication year was restricted to 2009–2019 (S1 Table). To identify additional articles, reference lists and grey literature sources including international and national surveillance databases and major academic websites were searched (S2 Table).

### Study selection

Articles retrieved from the literature searches were deduplicated and thereafter screened against predefined eligibility criteria following a two-stage screening process. First, titles/abstracts were screened, and second, full texts were screened. At both stages, screening was done independently by two reviewers, and discrepancies were solved by a third reviewer. Studies were included in the review if they reported on the incidence, seroprevalence, serotype distribution, hospitalisation, mortality, EFs, or direct and indirect costs of dengue in Thailand (S3 Table). Study design criteria were not applied to grey literature because methods and sources for data collection/analysis are not clearly or often reported for this publication type. For studies with multiple publications, the source with the most recent or complete dataset was included.

### Data extraction and synthesis

Relevant data were collected from each included publication onto a structured data extraction form. Data were extracted by one reviewer and cross-checked by a second reviewer. Any discrepancies were resolved by a third reviewer. Extracted data were descriptively synthesised. Data from national and regional surveillance sources were prioritised and supplemented with those from peer-reviewed publications where needed. For this reason, quality assessment was not performed because most of the included publications were from surveillance sources, and such assessment will not influence the data synthesis or certainty of the quality of evidence. No meta-analyses were conducted due to heterogeneity in the reported data. Costs were converted to 2019 USD using Thai consumer price index (CPI) [22].

## Results

**Fig 1:**
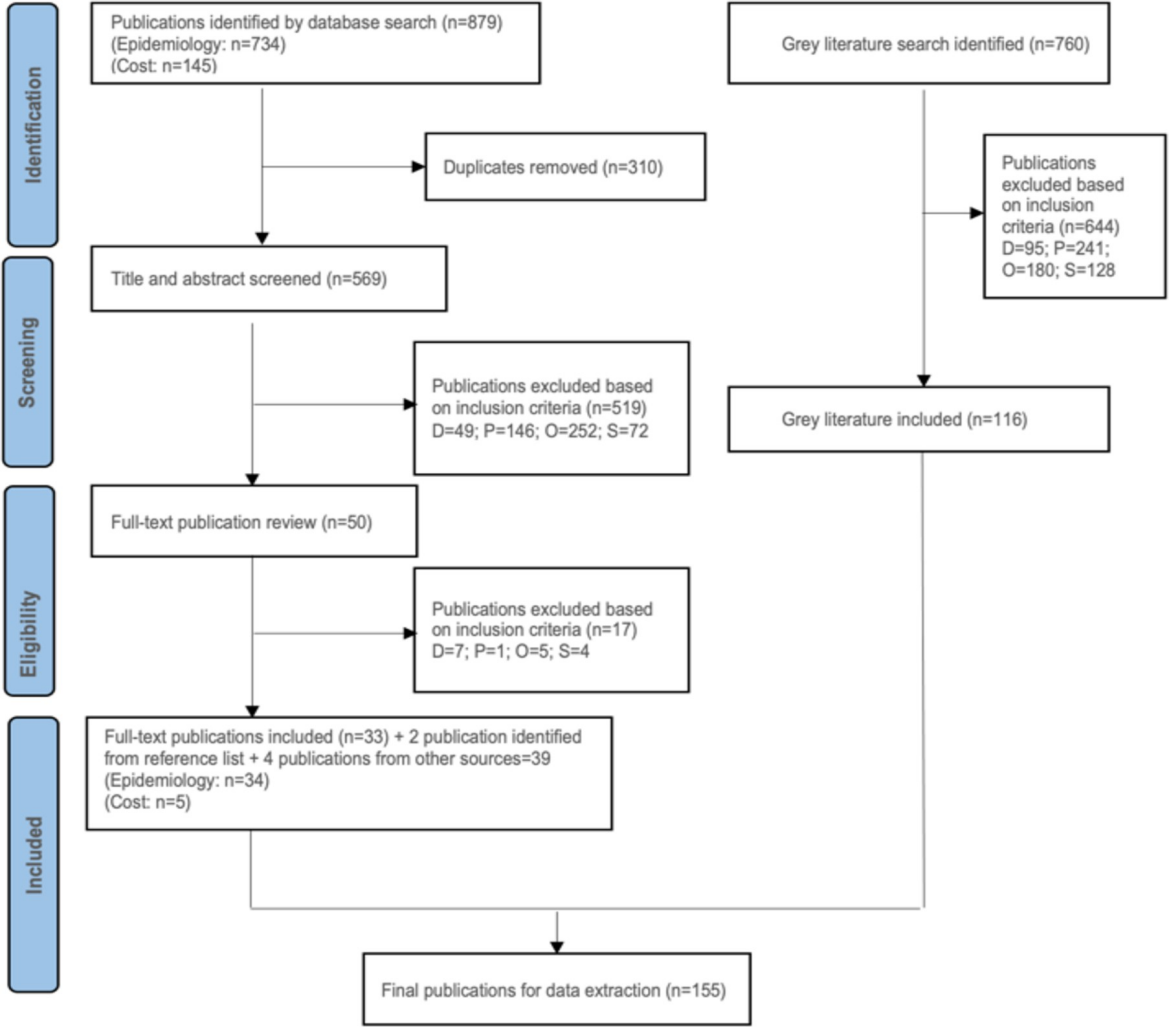
PRISMA flowchart for epidemiology and cost studies. D: duplicate; O: outcome; P: population; S: study design.

A total of 879 records were identified from the database search (734 from the epidemiology search and 145 from the cost search). Of these, 569 unique records were selected for title and abstract screening, of which 50 (48 from the epidemiology search and 2 from the cost search) were eligible for full-text screening. Thirty-three publications were selected for data extraction and inclusion into the review. Two additional publications captured from the review of reference lists were included in the review, resulting in 33 publications in the epidemiology search and two in the cost search. However, three publications identified in the epidemiology search also contained costs data and one publication in the costs search contained epidemiology data, yielding a total of five publications for the cost analysis and 34 publications for the epidemiological analysis (Figs 1, S1, and S2). Grey literature searches yielded a total of 760 articles, of which 116 were included in the review (Fig 1).

**Fig 2:**
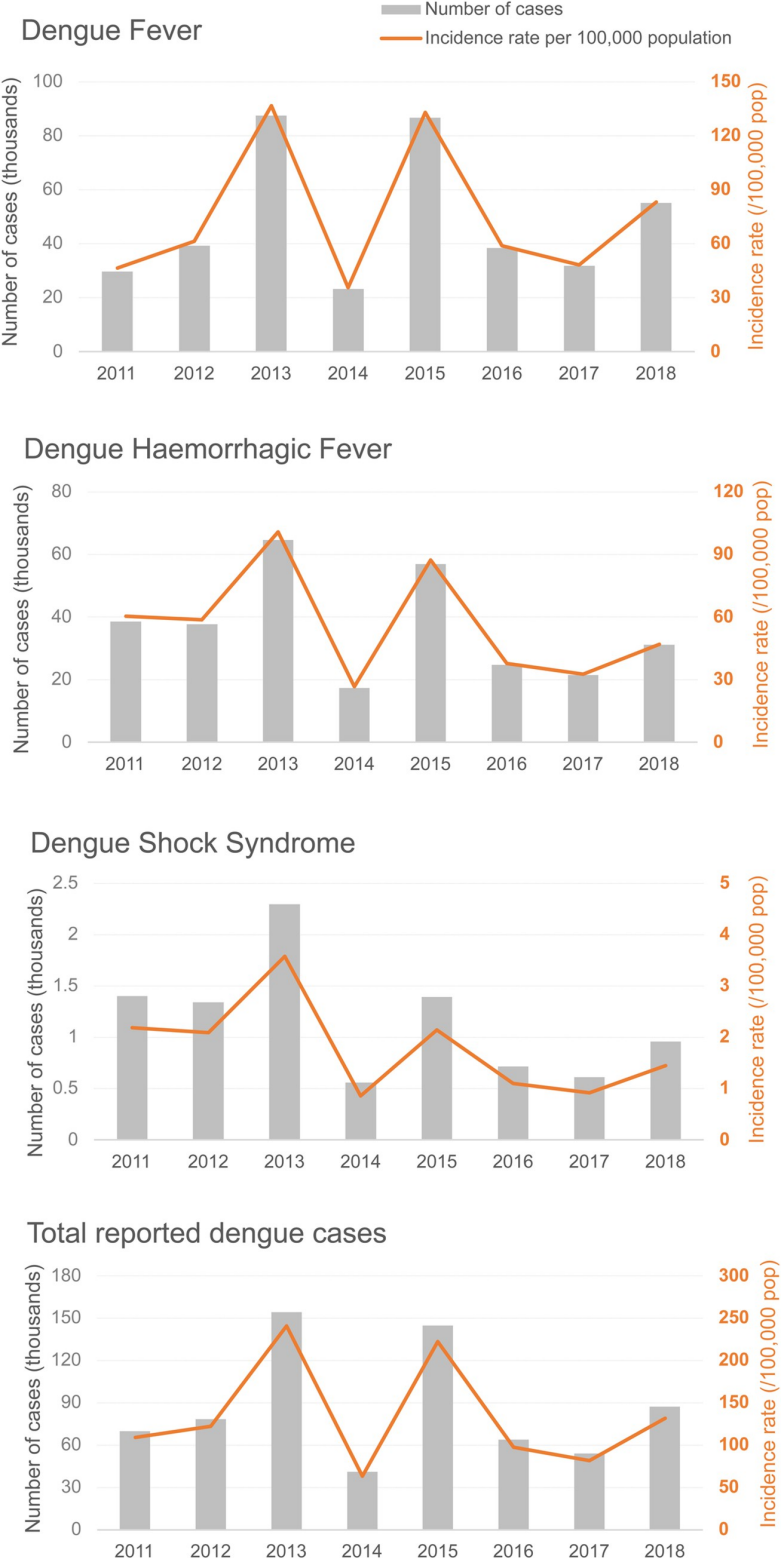
National number of cases and incidence rates of dengue by 1997 WHO case definition between 2011–2018; Total reported dengue cases represent dengue fever, dengue haemorrhagic fever, and dengue shock syndrome. Source: MoPH [12].

National incidence rates of dengue by case definition (DF, DHF, DSS) and age group were obtained from the MoPH reports and represent reported cases [12]. Overall, the annual incidence rates of DF for the period 2011–2018 varied from 35.67 to 136.56 per 100,000 population and were higher than those recorded for DHF (26.72 to 100.89 per 100,000 population), except in 2011 when the rate for DHF was greater than that for DF (60.39 vs. 46.52 per 100,000 population). Epidemics occurred every 2–4 years with over 130 cases per 100,000 population recorded for DF and over 80 cases per 100,000 population recorded for DHF in 2013 and 2015. As expected, annual incidence rates of DSS were significantly lower than both DF and DHF and ranged from 0.86 to 3.58 per 100,000 population. For all three case definitions, the lowest incidence throughout the review period was recorded in 2014 (Fig 2 and Table A in S4 Table) [12]. It should be noted that the incidence rates reported in this section were referred to as morbidity rates in the MoPH reports, and no data were available for 2019 at the time of this review.

### National incidence

**Fig 3:**
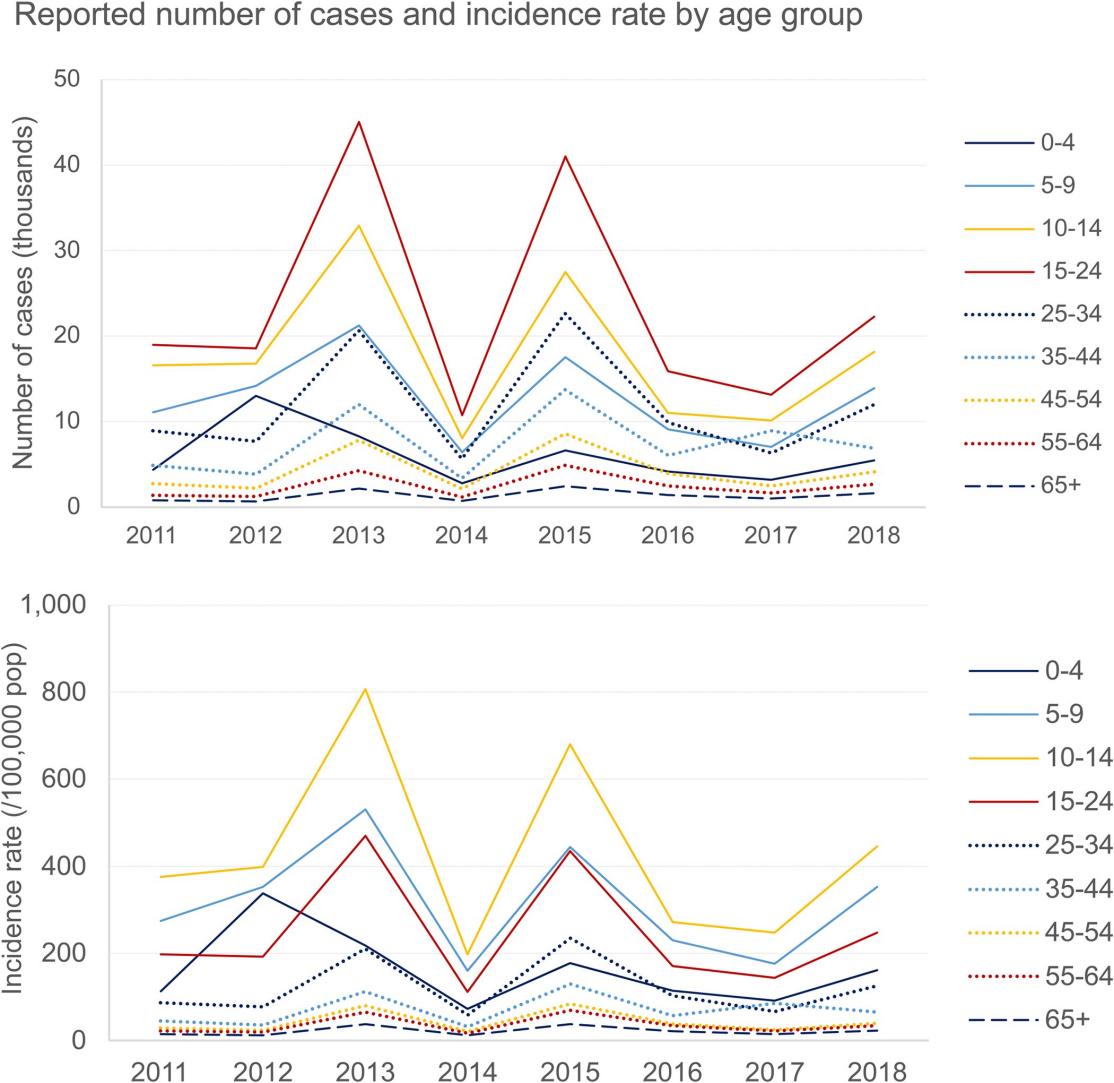
Reported number of cases and incidence rates of dengue per age group between 2011–2018; Source MoPH [12].

Nationally, most dengue cases reported between 2011–2018 were among 15–24-year-olds. However, the highest incidence rates for the period mentioned above were recorded in the 10–14-year-olds, followed by the 5–9-year-olds and then the 15–24-year-olds, except in 2012 when the rate was higher in the 0–4 age group than in the 15–24-year-olds (Fig 3 and Table B in S4 Table). Irrespective of case definition, the 5–9, 10–14, and 15–24-year-old age groups consistently contributed more than half of the total number of reported cases throughout the review period (Table C in S4 Table) [12].

**Fig 4:**
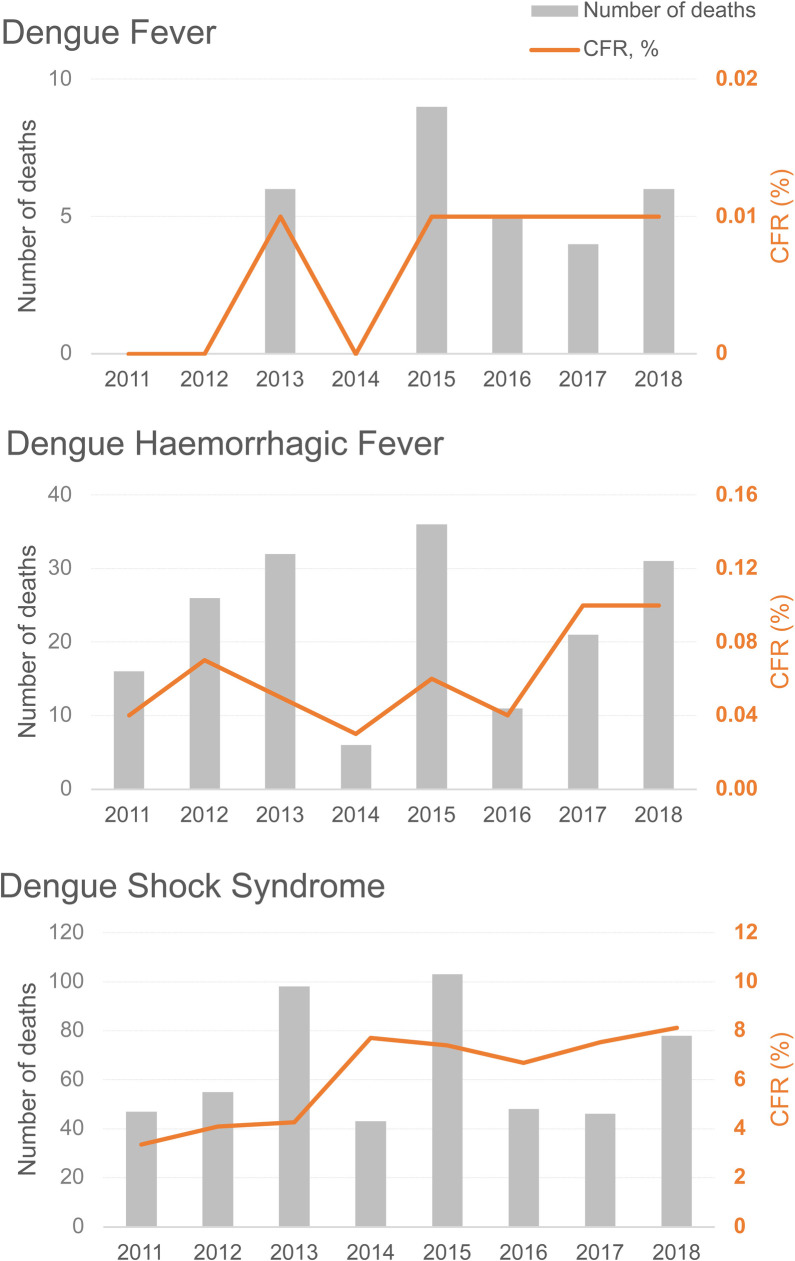
National number of deaths and CFRs by 1997 WHO case definition between 2011–2018; Source MoPH [12]. CFRs, case fatality rates. Note for dengue fever, no deaths were reported in 2011, 2012, and 2014.

Based on data obtained from the MoPH, the average hospitalisation rate for the total reported dengue cases from 2013–2018 was 63.3% (range 61.9%–65.7%), and in 2016, the rate was 75.3% for DHF and 52.9% for DF [12]. The total number of hospitalised cases was not reported in 2011, 2012 and 2019; and by case definition, data were reported only in 2016 (Table A in S5 Table). Lumbiganon et al. retrospectively analysed nationwide hospitalisations in which a total of 191,016 cases were reported in 2010, and the majority were attributed to DHF (107,001 cases) compared to 74,297 cases for DF patients. Overall, the hospitalisation rate was highest in the 13–18-year-olds (8.9 admission rates per 1,000 population, n = 51,567) and lowest in the 80+ age group (0.2 admission rates per 1,000 population, n = 240). Although for DF, the total number of hospitalisations was highest in the 6–12-year-olds (S6 Table) [23]. Using data from multiple sources, Shepard et al. estimated 1,687,363 dengue infections in Thailand in 2013, of which 337,378 cases were hospitalised (20%) [17]. Annual mortality rates of dengue per 100,000 population between 2011–2018 were ≤0.01 for DF, between 0.01 and 0.06 for DHF, and between 0.06 and 0.09 for DSS. The highest number of deaths and case fatality rates (CFRs) were reported in the DSS group, and the age groups most affected were the 15–24-year-olds, followed by the 10–14-year-olds. The overall pattern of deaths aligned with the major outbreaks that occurred in 2013 and 2015, although for DSS, the CFRs peaked in 2014 (7.7%) and 2018 (8.13%) (Fig 4 and Tables A and B in S5 Table) [12]. Lumbiganon et al. reported the combined mortality rate of DF and DHF for 2010 as 0.3 per 100,000 population. Categorised by age, the mortality rate was highest in the 6–12-year-olds (0.8 per 100,000 population) and lowest in the 25–79-year-olds (0.1 per 100,000 population) [23]. Shepard et al. estimated 246 dengue deaths in 2013, of which 163 were in children (<15 years) and 83 were in adults (> = 15 years) [17].

### Regional incidence

**Fig 5:**
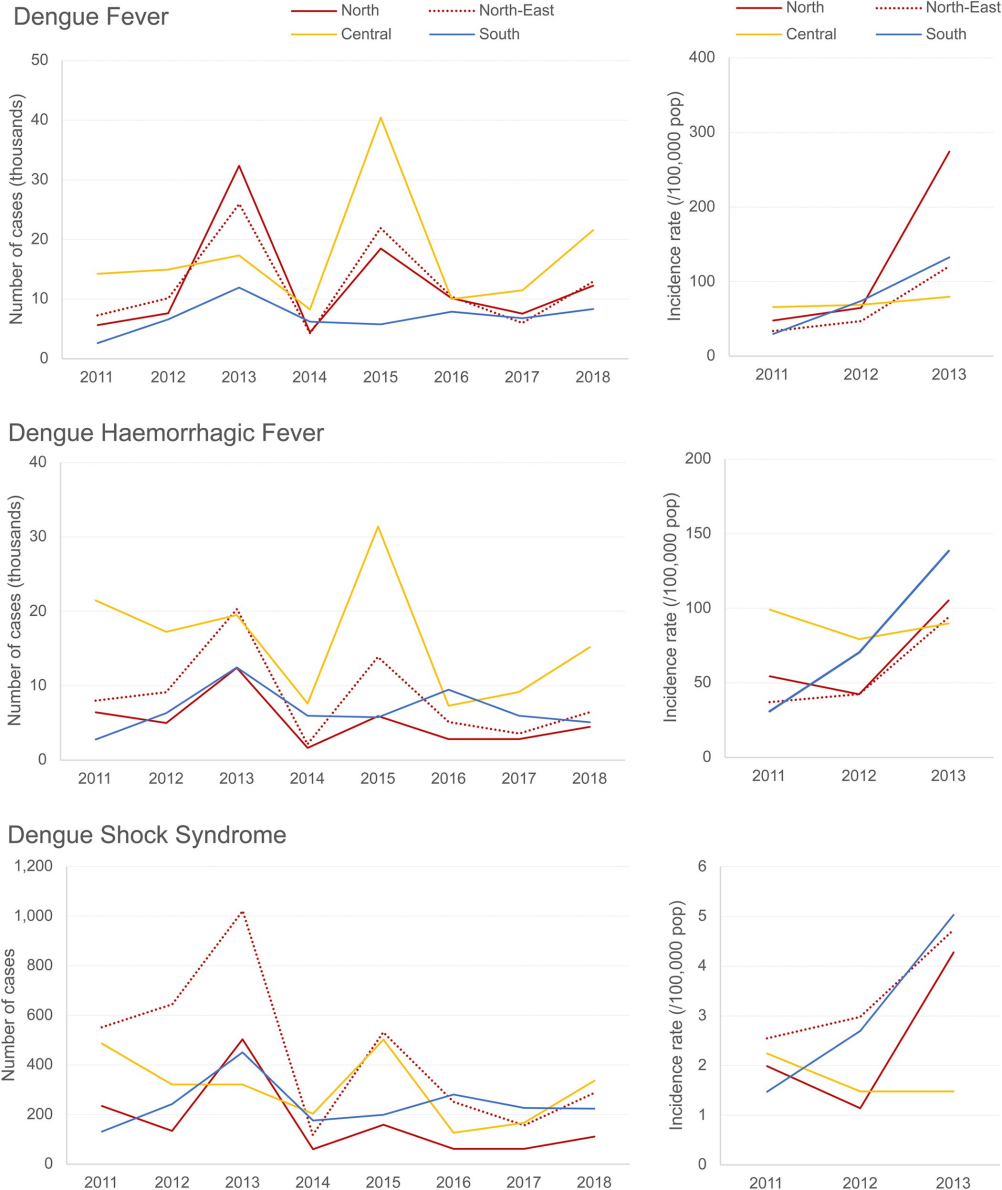
Regional number of cases and incidence rates of dengue by 1997 WHO case definition between 2011–2018; Source MoPH [12].

Regional incidences of dengue were mainly obtained from the MoPH reports, which reported the number of cases and incidence rates from 2011–2013 and only the number of cases from 2014–2018 [12]. During the review period, the Central region registered the highest number of DF (8,300–40,438) and DHF (7,590–31,414) cases each year (S7 Table). However, in 2013 and 2016, the highest number of cases were observed in the North (32,244 cases) and Northeast (10,400 cases) regions for DF and in the Northeast (20,287 cases) and South (9,464 cases) regions, respectively for DHF. The burden of disease fluctuated by region for DSS, with the highest number of cases reported in the Northeast between 2011–2013 and 2015, the Central region in 2014 and 2018, and the South region in 2016–2017. Similarly, incidence rates by case definition varied across the regions for the period 2011–2013 and the highest rate of 274.49 per 100,000 population was registered in 2013 for DF in the North region (Fig 5 and S7 Table). Furthermore, provincial dengue incidence also varied each year [12]. Seven additional publications reported provincial incidence (Tables A and B in S8 Table) [4,13,14,24–29]. Although the studies varied in terms of dengue case definitions and study methodology, the published data for total reported cases for all ages from three studies with the largest study periods were consistent with those reported by MoPH [4,13,26]. Nealon et al. calculated adjusted dengue incidence per 100,000 person-years, while Wichmann et al. used dengue incidence derived from laboratory-confirmed cases in prospective cohort studies. Both studies indicated notably higher dengue incidence than national surveillance data (Tables A and B in S8 Table) [14,27].

Regional incidence by age group was reported by six publications, [4,13,14,25,27,28]. In the period between 2011–2013, the 2–4-year-olds had the highest incidence of febrile episodes (116.8 per 100 person-years), while the 5–8-year-olds had the highest incidence of virologically confirmed dengue (VCD) (6.8 per 100 person-years). Incidence rates for DHF were similar in the age groups 5–8 and 9–12-year-olds (0.4 per 100 person-years) [25]. Wichmann et al. reported the incidence of laboratory-confirmed dengue in Ratchaburi and Kamphaeng Phet provinces between 2004 and 2007 and showed that the 0–4-year-olds had the highest incidence of dengue (44.8 per 1,000) in 2007 [27]. In a prospective cohort study of primary school children aged 3–13 years in Ratchaburi, the incidence of dengue per 100 person-year ranged from 1.77 to 5.74, with the average incidence over four years (2006–2009) reported as 3.6% [28]. In Phanitchat et al., cases of dengue in Khon Kaen Province were most frequently reported in the 5–14-year-olds (51.1%), followed by the 15–29-year-olds (33.1%), and then the 30–44-year-olds (6.2%) between 2006 and 2016. The proportion of cases among older age groups (>15 years) increased from 20% in 2006 to >50% in 2016 [4]. Tanayapong et al. reported similar age group shifts towards older age groups (>15 years) throughout the period 2000–2010 for Ratchaburi, although rates of dengue were consistently high in children <15-year-olds [13].

Regional CFRs differed for DF, DHF, and DSS during the review period, with higher rates reported for DHF than for DF (S9 Table). The highest CFRs for DF were reported in Pattani in the epidemic year 2015 (South, 0.68%) and in Samut Sakhon in 2016 (Central, 0.68%); for DHF, in Uttaradit in 2016 (North, 4.17%). Wide CFRs ranging from 1.36%–100% were recorded for DSS [12,13,29].

For regional hospitalisation, data were reported by eight publications but the differences in age, case definition, and period classification make data summarisation challenging (S10 Table) [14,25,27,29–33].

### Seasonality

**Fig 6:**
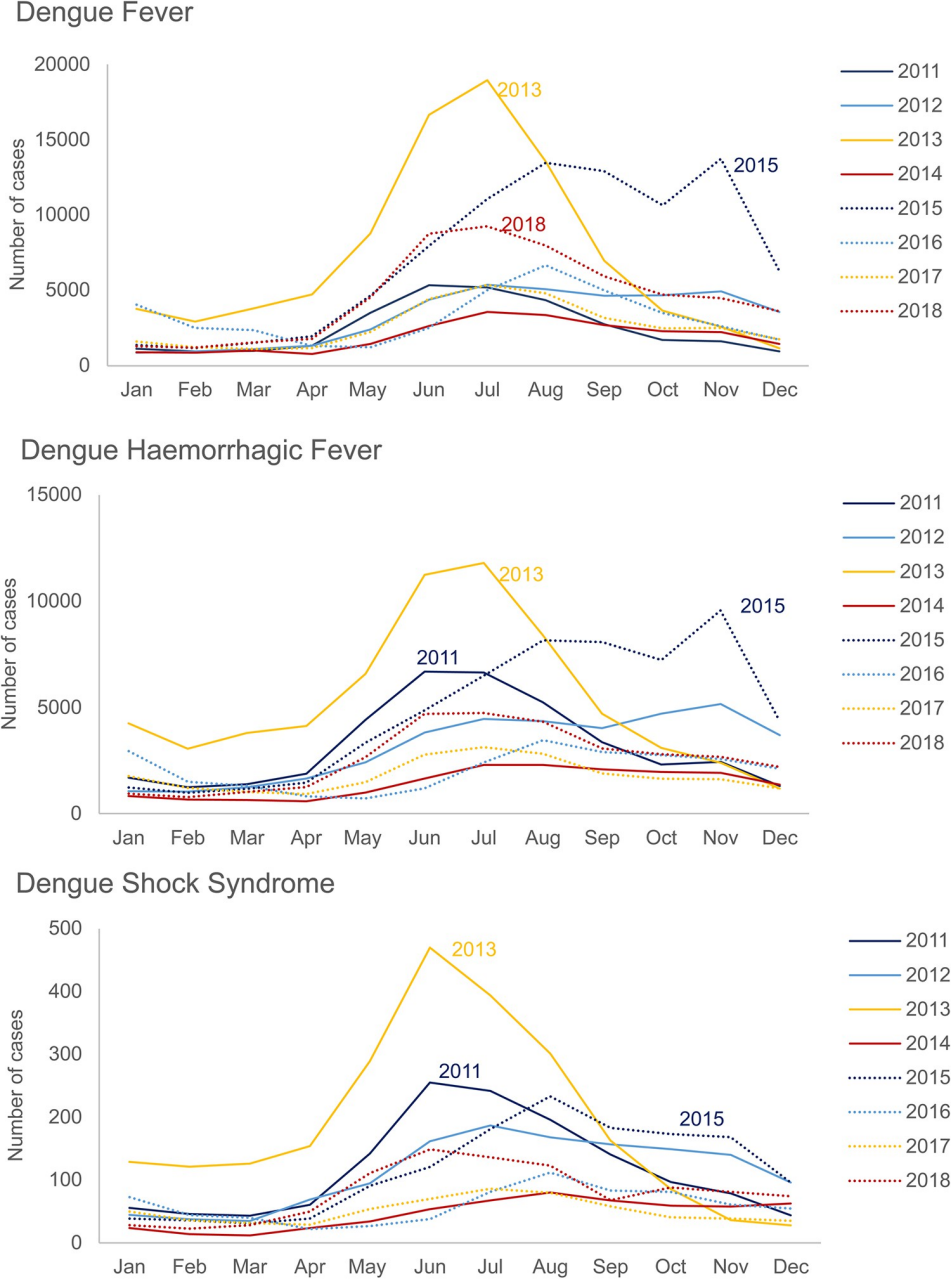
Number of dengue cases by 1997 WHO case definition per month between 2011–2018; Source MoPH [12].

National surveillance reports indicate that the annual peak of dengue cases in the period 2011–2018 was between May and August, except in 2015, when the peak extended to November (Fig 6) [12]. The seasonal peak of dengue cases was confirmed by five other publications that reported the highest incidence rates of dengue during the rainy season from mid-May to mid-October (S11 Table) [4,13,26,31,34].

### Seroprevalence

A total of sixteen publications reported the seroprevalence of dengue across different populations and provinces using various testing methods [25,28,29,35–47]. Overall, the seroprevalence varied between 52%–95% [25,28,29,33,35–37,39,41,46,47]. In the age groups, 1–10 years old, 11–20 years old, 21–25 years old, and >25 years old, seroprevalence across the studies ranged between 7.8%–72%, 18.0%–84.2%, 27.3%–57.1%, and 42.8%–100%, respectively. (S12 Table). High rates were also seen in younger age groups between 2–8 years using data from two clinical trials (CYD14 from June to December 2011 57.7% and CYD23 from February 2009 to February 2010 65.8%) [42], 6–10 years (61%) [39], 10–14 years (63.2%), 15–19 years (74.2%) [29], and 13–16 years (84.2%) [25].

### Serotype distribution

Available data showed that all DENV serotypes co-circulate in Thailand but to different extents. From the Department of Disease Control, the most common serotype in Thailand in 2014 was DENV3 (35.3%), followed by DENV4 (30.8%), DENV1 (28.8%), and DENV2 (5.13%) [48]. From published studies reporting data for different provinces, the most predominant serotype reported between 2004–2009 was DENV1 [31,33,46,49,50], but in 2007, dominance shifted to DENV4 in Bangkok (Central) and DENV1 and DENV2 cocirculated in Ratchaburi (Central) [31,50]. Between 2010 and 2011, DENV2 was the most dominant serotype reported in Chiang Mai, Lumphun, Lumpang, Mae Hong Sorn (all North), and Nong Khai (Northeast) (S13 Table) [29,46,50]. Both DENV2 and DENV4 were equally predominant in 2004 in Si Sa Ket (Northeast) [51]. In a study in Southern Thailand, the most prevalent serotype reported in an epidemic season (year not reported) was DENV2 (64%), followed by DENV3 (24%), and the least common was DENV4 (4%) [52]. Serotype distributions in 2013 and between 2015–2019 were not reported at the time of this review. Details on seroprevalence by serotype are summarised in S14 Table.

### Expansion factor

Three publications reported EFs to account for underreporting of dengue in Thailand, but the results were highly varied (Table 1). In general, EFs differed by treatment setting (inpatient or outpatient care), case definition, and region. Wichmann et al. reported an EF of 3.7 for outpatient dengue cases compared to 1.9 for inpatient cases in the 5–12-year-olds in Kamphaeng (North). However, in the 0–14-year-olds in Ratchaburi (Central), the EF was greater for inpatient cases (3.3) than for outpatient cases (1.0). A national average EF of 8.7 was estimated for overall symptomatic dengue cases and 2.6 for inpatient cases [27]. Based on the ratio between the incidence of VCD and suspected dengue cases for the same age group (9–14 years), area, and period (2011–2013) during the CYD14 trial, Coudeville et al. used an EF of 6.2 to account for dengue cases in all age groups [53]. Using a similar method, Nealon et al., reported the highest EFs: 12 for VCD cases, 8.8 for clinically diagnosed dengue, and 8.6 for clinically diagnosed and VCD cases [14]. Both studies were based on the same trial and were more robust methodologically, since the EFs were obtained by comparing the incidences from the active surveillance of the control arm of the CYD14 trial with the incidence rates obtained from the national passive surveillance systems.

**Table 1:** Expansion factors by locality, patient subgroup/age, and healthcare setting. CI, confidence interval; EF, expansion factor; ID, incidence density; IRs, incidence rates; NR, not reported; VCD, virologically confirmed dengue.

Author, year	Locality	EF data source	EF data source/methodology	Year	Subgroup or age group	Inpatient cases	Outpatient cases	Total cases
Wichmann et al. (2011) [27]	Kamphaeng Phet	Children <15 years from the field sites and provinces of the cohort study	**Age-specific inpatient multiplication factors:** Dividing the incidence of laboratory-confirmed inpatient dengue cases in the cohort by the reported incidence of dengue inpatient cases in the same province and year **Age-group outpatient multiplication factors:** Dividing the number of laboratory-confirmed dengue outpatients by dengue inpatients in the cohort by year	2004	5–9 years	1.8	4.4	NR
					10–12 years	0.7	5	
				2005	5–9 years	0.3	9	
					10–12 years	4	3.3	
				2006	5–9 years	2.6	3.9	
					10–12 years	6	2	
				2007	5–9 years	1.3	4.5	
					10–12 years	0.5	6.5	
				2004–2007	5–12 years	1.9	3.7	
	Ratchaburi			2006	0–4 years	2.5	NR	
					5–9 years	2	NR	
					10–14 years	3.8	NR	
				2007	0–4 years	10.3	1.1	
					5–9 years	3.1	0.9	
					10–14 years	2.4	1.4	
				2006–2007	0–14 years	3.3	1	
	National			2003–2007	<15 years	2.6	NR	8.7
Coudeville et al. (2016) [53]	National	EFs for 9–14-year-olds used in CYD14 trial	Ratio between incidence of VCD and suspected dengue cases for the same age group, area, and period during phase III trials and reported incidence were used to correct routine surveillance data in all age groups	2011–2013	9–14	NR	NR	6.2 (95% CI: 4.6,8.0)
Nealon et al. (2016) [14]	National	EFs for 0–14-year-olds used in CYD14 trial	Calculated by dividing the adjusted ID captured during CYD14 for each case definition by the IRs reported by the national passive surveillance systems at each geographical unit. For calculating 95% CIs, IRs from surveillance systems were considered known data without variability	06/2011–12/2013	VCD case	NR	NR	12.0 (95% CI: 8.6,16.2)
					Clinically diagnosed and VCD cases	NR	NR	8.6 (95% CI: 5.9,12.2)
					Clinically diagnosed dengue cases	NR	NR	8.8 (95% CI: 6.1,12.5)

EF: Expansion factors, numbers by which the reported cases are multiplied to provide accurate estimates of incidence, are derived by comparing cases identified by active surveillance to passive surveillance.

### Dengue costs: direct medical and non-medical costs

Over the review period, five studies reported the costs of dengue in Thailand, but the data were limited. All costs were converted to 2019 USD using Thailand specific CPI except one study that did not report the costing methods or valuation year. Chanthavanich et al. reported the private direct out-of-pocket cost of dengue in Bang Phat district of Ratchaburi (Central) as Thai Baht (THB) 1,123 [54].

Shepard et al. reported the direct costs of hospitalised, ambulatory, and non-medical dengue cases from a nationwide perspective. In all three settings, direct medical and non-medical costs were higher than indirect costs associated with productivity. Direct costs of hospitalisation per case (USD 638.50) were also greater than ambulatory costs per case (USD 159.62). The overall average cost per dengue case was USD 260.96. After adjusting for underreporting, the total aggregated annual dengue cost was estimated at USD 440.3 million, with direct cost (USD 366.1 million) contributing more than 80% and hospital cases (USD 233.7 million) constituting nearly 64% of the total direct cost [17]. This is supported by Suaya et al. and Lee et al., who analysed the economic burden in Khon Kaen (Northeast) and Ratchaburi (Central) provinces [55,56]. The average total cost per case was estimated at USD 40.69 and the cost per day was USD 7.12 [55].

Among hospitalised patients, Tozan et al. showed that the household costs of dengue in Chachoengsao (Northeast) increased with disease severity, with a higher cost reported for DHF than for DF in the age groups <15 years (USD 170.70 vs. USD 157.67) and ≥15 years (USD 232.09 vs. USD 175.73). Regardless of severity, the cost was greater in adults than in children [32].

### Societal impact and productivity cost of dengue

Shepard et al. in their nationwide economic cost analysis showed that productivity costs of hospitalisation per case (USD 54.94) were greater than ambulatory costs per case (USD 13.47) in Thailand [17].

Furthermore, three studies reported limited data on the societal impact of dengue in Thailand. The description of productivity cost parameters applied in each study is provided in the S15 Table. As expected, DHF patients had a higher number of days lost at work (7.6 ± 3.1 days vs. 6.6 ± 3.6 days) or school (8.6 ± 5.1 days vs. 6.3 ± 3.5 days) than those with DF [32]. Overall, the longest number of sick days were among inpatients (6.0 to 10.8 days) and those with severe disease (DHF, 10.0 to 10.5 days). The average hospital stay for all patients was 4.9 ± 3.3 days and 4.6 ± 1.3 days for paediatric patients, respectively [32,55,56]. In the context of disease severity, the average productivity cost (USD 28.48) of DF for outpatients was higher than direct medical (USD 7.12) and non-medical costs (USD 5.09) [55].

## Discussion

The review describes the recent trends in the epidemiology and costs of dengue in Thailand. Overall, the annual incidence of dengue varied from 35.7 to 136.6 per 100,000 population for DF, from 26.7 to 100.9 per 100,000 population for DHF, and from 0.86 to 3.58 per 100,000 population for DSS, but the data were relatively stable throughout the review period except in the epidemic year of 2013 and 2015, which had the highest incidence [12,34]. The trends are similar to a previous review for Thailand that reported an average annual dengue incidence of 115 cases per 100,000 population for the period 2000–2011 [3].

From a regional perspective, the incidence of dengue varied year to year as observed at the national level (12). Across the review period, dengue cases were consistently high in the Central region as shown previously by Limkittikul et al., [3]. Overall, data from this study and previous studies depict a stable trend for national and regional incidence for the period 2000–2018 [3,12]. Nonetheless, the disease burden data should be interpreted cautiously, as national surveillance data represent reported cases and are not adjusted for underreporting.

The seasonal impact of dengue was most evident during the rainy season, with most cases occurring from mid-May to mid-October each year [4,12,13,26,31,34]. This coincides with the monsoon season. The seasonal hikes together with the high burden of dengue in Thailand could overwhelm the healthcare systems and adversely impact the management and outcomes of other diseases [57].

Although the incidence of DHF and DSS was stable, the number of deaths in both disease groups increased throughout the review period, except in 2014 and 2016/2017 when it declined. The annual CFRs for the period ranged from 0.03%–0.1% for DHF and 3.35%–8.13% for DSS and surprisingly, 2014, which recorded the lowest number of deaths, also reported the second highest CFR for DSS during the review period. Throughout the period, the highest numbers of deaths were reported in the 15–24-year-olds followed by the 10–14-year-olds. In the literature review from Limkittikul et al., no deaths were recorded for DF between 2003 and 2011 [3]. However, in this review, deaths were reported in 2013 and consistently between 2015–2018. The mortality data should be carefully interpreted considering underreporting or misreporting of dengue-related deaths.

From a national perspective, the age groups most impacted by dengue during the review period were the 10–14-year-olds (197.7 to 806.9 per 100,0000 population), the 5–9-year-olds (159.8 to 530.1 per 100,000), and the 15–24-year-olds (112.3 to 470.3 per 100,000 population), implying that in Thailand, dengue is primarily a disease of children and young adults as previously reported by Limkittikul et al. for the period 2000–2011 [3].

Hospitalisations due to dengue were high, ranging from 61.9%–65.7% at the national level. Higher rates, which varied by case definition and diagnostic status were observed at the regional level [12,17,23,25,29]. The majority of hospitalised cases were school children aged 6–18 years [23], and the rate was expectedly higher for DHF than for DF [12,23].

It is well documented that the four DENV serotypes co-circulate in Thailand [4, 58]. At the provincial level, DENV1 and DENV2 were mostly isolated [29,31,43,46,49,50,52,59, 60], with the DENV2 known to cause severe disease. The MoPH did not report national and regional serotype distributions. Data from publications were challenging to summarise due to variation in the predominant circulating serotype at the province/district level, and the distributions were not reported yearly [28,29,33,39,44,46,49,50,52,59,60]. The paucity of information highlights the need to continuously monitor the shift in serotype dominance, as it may help to forecast future major outbreaks [61].

National and regional seroprevalence data were not reported by the MoH. However, publications based on specific provinces and districts in Thailand showed diverse high seroprevalence rates, with one study conducted in Ayutthaya, Lop Buri, Narathiwat, and Trang, reporting seropositivity as high as 79.2% [41]. High rates of up to 84% were reported in the age groups between 2 and 19 years old, further highlighting the burden of dengue in children in Thailand [25,29,39,42]. Secondary dengue infections were more prevalent than primary infections, with studies from Yoon et al. (81.8%–97.4% vs. 0%–18.2%) and Buddhari et al. (85.9% vs. 2.8%) indicating high endemicity [33,45].

Dengue was also shown to impose a significant economic burden on the healthcare systems, households, and society, but the true burden may be underestimated due to underreporting and misreporting inherent in passive surveillance systems. Given that dengue is often self-managed by patients and that an outpatient visit or hospitalisation is the primary point of recording symptomatic dengue cases, the official reported cases represent only a fraction of the true burden of dengue. Estimation of the true burden is further skewed by regional and socioeconomic disparities and a lack of continuous reporting. Several studies identified in this systematic review highlight substantial underreporting of dengue cases in the national surveillance system, thus emphasising the need to adjust reported cases using EFs based on different settings and contexts to account for underreporting. In Nealon et al., substantial underreporting of symptomatic dengue cases was observed from passive surveillance systems in Thailand, resulting in the use of EFs of 8.6 to 12, depending on the case definitions. Similarly, Wichmann et al. highlighted the under-recognition of the burden of dengue by the passive surveillance systems in Thailand, with total symptomatic dengue cases 8.7-fold higher and inpatient cases 2.6-fold higher than recognised [14,27]. A previous economic systematic analysis estimated an EF of 8.5 for total dengue episodes in Thailand for the period 2001–2010 [62]. The paucity of national-level economic studies limits the estimation of true dengue economic burden in Thailand. Direct medical and non-medical costs may vary across the country. Societal and productivity costs are further challenged by the parameters included in the economic evaluations. Among the five studies identified in this review, three regional studies analysed productivity costs by considering data on school absenteeism, lost days at work, and days lost by either the patient or the caregiver during the illness [32,55,56]. Most of the studies only considered absenteeism from paid work and did not consider economic losses associated with lost unpaid work and costs incurred by informal caregivers. Altogether, economic burden is likely underestimated [63].

The strength of this review lies in the comprehensive evaluation of both the epidemiology and costs of dengue in Thailand over the past decade. However, several limitations exist. First, the literature search is liable to publication bias because publicly available studies/data were primarily identified. Second, incidence and mortality data were mostly obtained from grey literature sources and where possible, complemented with data from peer-reviewed studies. Further, the restriction to specific provinces or districts by most of the studies, as well as varying case definitions and dates of data collection hampered adequate comparison. Third, no publications reported seroprevalence data between 2017 and 2019; likewise, for serotype distribution, no data was available from 2015 to 2019. Fourth, in the cost analysis, only five studies were identified, and none stratified results by region and healthcare sector (private or public). Furthermore, variation in costs was observed due to the heterogeneity in methodological choices, which directly affects the comparison between the studies. Besides reporting on loss of working days, the broader macroeconomic impact of dengue has been overlooked. No studies included in the SLR analysed the impact of dengue on foreign direct investment or the income lost due to decreased tourism during seasonal dengue peaks and epidemics [32,55,56]. The true extent of direct and indirect costs incurred by lower socioeconomic sections poses a profound equity challenge [64]. Dengue epidemics are unpredictable and have been noticed in previously unaffected areas, with the spread of the dengue vector due to global climate changes, ineffective vector control methods, inconsistent surveillance, and travel [65,66]. Dengue vaccines could potentially reduce epidemiological and economic burden of dengue in Thailand [67]. Therefore, there is a need to evaluate effective dengue vaccination strategies and their cost-effectiveness to complement other preventive measures [68].

## Conclusion

Limited evidence is available for an accurate estimation of the dengue incidence and economic burden in Thailand. The overall burden of dengue in Thailand is high, with incidence rates varying year to year and national epidemics occurring every 2–4 years. Regardless of case definition, children are most affected, particularly the 5–14-year-old age group. Peak incidence corresponds with increased rainfall, and the impacts on terms of hospitalisations, deaths, and cost increase with disease severity. Economic and societal effects are substantial but underestimated due to underreporting and misreporting of cases. Therefore, it is essential to use EFs to adjust for underreporting so that the most effective public health interventions can be developed and implemented. Instead of limiting active surveillance to outbreak periods and settings only, continuous monitoring of dengue cases and serotypes is suggested to prevent, predict, and control future outbreaks. In view of the high cost and limited efficiency of vector control measures, further emphasis should be placed on evaluating vaccination strategies in integrated dengue management programmes to benefit broader population groups and alleviate socioeconomic disparities.

## Supporting information

S1 Fig PRISMA diagram for epidemiology studies. (TIF)

S2 Fig PRISMA diagram for cost studies. (TIF)

S1 PRISMA Checklist PRISMA 2020 checklist. (DOCX)

S1 Table OVID search strategy. (XLSX)

S2 Table Literature sources. (XLSX)

S3 Table Eligibility criteria. (XLSX)

S4 Table Table A. MoPH National annual incidence of dengue by case definitions. Table B. MoPH number of dengue cases (DF+DHF+DSS) calculated. Table C. MoPH National annual incidence of dengue by age and case definitions.
(XLSX)

S5 Table Table A. MoPH national mortality, CFR, and hospitalisations from 2011–2018. Table B. MoPH number of deaths for DSS by age group from 2011–2018.
(XLSX)

S6 Table National hospitalisations and admission rates by age. (XLSX)

S7 Table MoPH regional incidence for DF from 2011–2013. (XLSX)

S8 Table Table A. Regional Incidence by dengue case definition from Nealon et al. 2016.Table B. Regional Incidence by dengue case definition from seven publications.
(XLSX)

S9 Table MoPH regional mortality and CFR from 2011–2018. (XLSX)

S10 Table Summary of hospitalisations due to dengue based on eight studies. (XLSX)

S11 Table Seasonal incidence of dengue. (XLSX)

S12 Table Seroprevalence of dengue. (XLSX)

S13 Table Serotype distribution. (XLSX)

S14 Table Seroprevalence of dengue by serotypes. (XLSX)

S15 Table Societal impact of dengue. (XLSX)
