# Supplementary figures and images for: Epidemiology and costs of dengue in Thailand: A systematic literature review
Source: PLoS Negl Trop Dis. 2022 Dec 19;16(12):e0010966. doi: 10.1371/journal.pntd.0010966 (PMC9810168; doi:10.1371/journal.pntd.0010966)

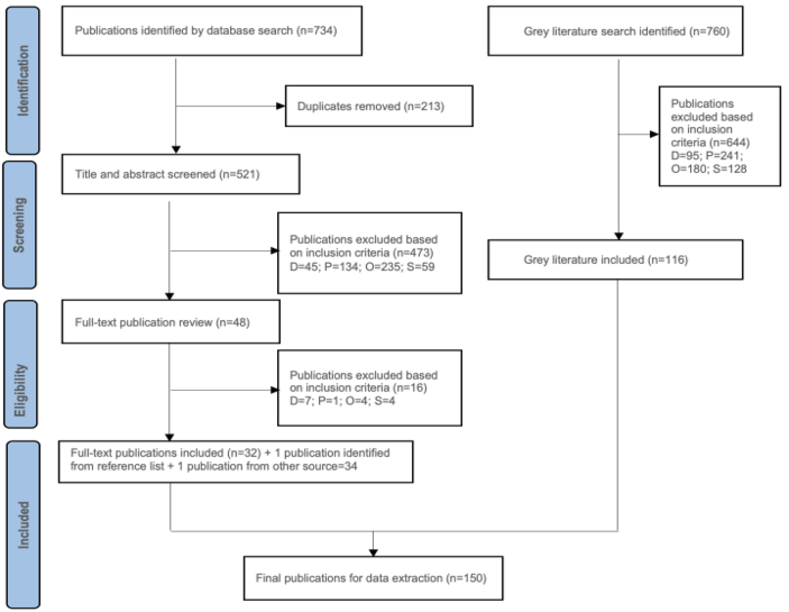

Supplement: S1 Fig — (TIF) [file pntd.0010966.s001.tif]

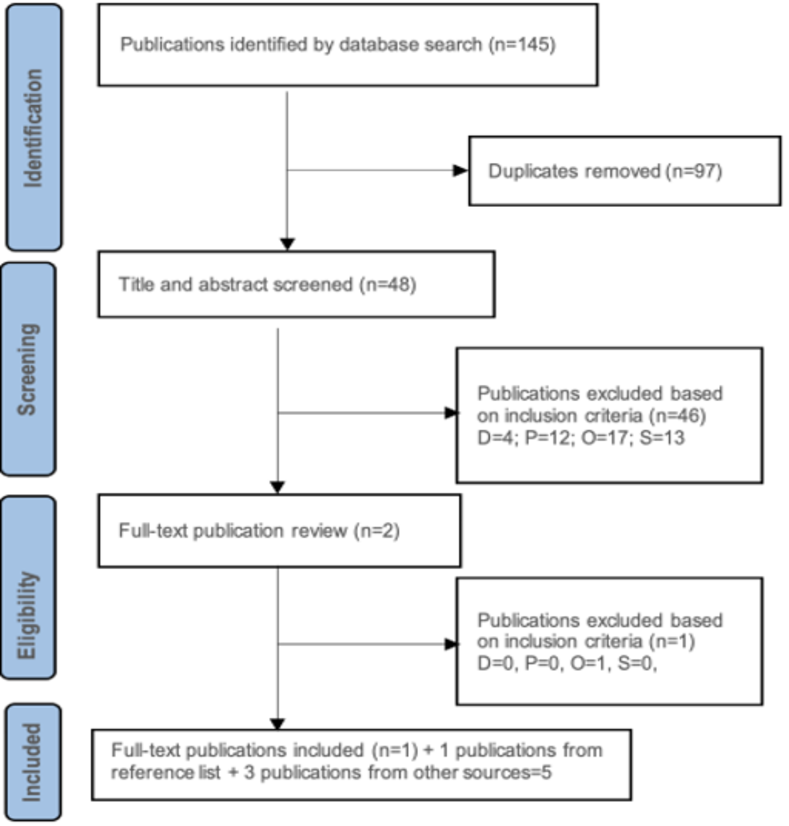

Supplement: S2 Fig — (TIF) [file pntd.0010966.s002.tif]
